# Influence of *CYP4F2*, *ApoE*, and *CYP2A6* gene polymorphisms on the variability of Warfarin dosage requirements and susceptibility to cardiovascular disease in Jordan

**DOI:** 10.7150/ijms.51546

**Published:** 2021-01-01

**Authors:** Laith N. AL-Eitan, Ayah Y. Almasri, Adan H. Alnaamneh, Hatem A. Aman, Nasr N. Alrabadi, Rame H Khasawneh, Mansour A. Alghamdi

**Affiliations:** 1 Department of Applied Biological Sciences, Jordan University of Science and Technology, Irbid 22110, Jordan.; 2 Department of Biotechnology and Genetic Engineering, Jordan University of Science and Technology, Irbid 22110, Jordan.; 3 Department of Pharmacology, Faculty of Medicine, Jordan University of Science and Technology, Irbid 22110, Jordan.; 4 Department of Hematopathology, King Hussein Medical Center (KHMC), Royal Medical Services (RMS), Amman 11118, Jordan.; 5 Department of Anatomy, College of Medicine, King Khalid University, Abha 61421, Saudi Arabi.; 6 Genomics and Personalized Medicine Unit, College of Medicine, King Khalid University, Abha 61421, Saudi Arabia.

**Keywords:** Warfarin, cardiovascular disease, *APOE*, * CYP4F2*, and *CYP2A6*

## Abstract

Cardiovascular diseases are among the leading causes of death worldwide. Many of those diseases require treatment with warfarin, an anticoagulant that has a large high inter and intra-variability in the required doses. The aim of this study is to find if there are any associations between rs2108622 of *CYP4F2*, rs7412 and rs405509 of *ApoE*, and rs1801272 of *CYP2A6*, and CVD and warfarin dose variability. The selected genes and their polymorphisms are involved in many GWAS associated with cardiovascular disease and variability in warfarin treatment. The study sample consisted of 212 Jordanian Cardiovascular patients and 213 healthy controls. DNA was extracted and the Mass ARRAY™ system was used to genotype four selected SNPs within three genes (*CYP4F2*, *ApoE,* and *CYP2A6*). Only one out of the four selected SNPs (*ApoE* rs7412 SNP) was found to be associated with the risk of cardiovascular disease. Also, this SNP showed significant differences in warfarin initial doses. *CYP2A6* rs1801272 SNP was found to be associated with warfarin sensitivity during the initiation phase of therapy and with warfarin responsiveness and INR measurement during the stabilization phase of therapy. This study improves the current understanding of the high inter and intra-variabilities in response to warfarin, including the variety of dosing requirements and the susceptibility to cardiovascular disease in the Jordanian Arab population. Further study on a larger sample and in different ethnic groups could help in improving our understanding of warfarin's pharmacogenetics and its application in personalized medicine.

## Introduction

Heart disease is a term commonly referred to as cardiovascular disease (CVD), which defines several disorders that affect the heart and blood vessels, including Coronary heart disease (CHD), Myocardial Infarction (MI), deep vein thrombosis, pulmonary embolism, and rheumatic heart disease 1. According to the World Health Organization (WHO), CVDs are the leading cause of death worldwide. In addition, more than three-quarters of CVD deaths occur in developing (low and middle-income) countries, such as Jordan 2. Therefore, in order to reduce the burden of CVD in these countries, we must struggle to combine our knowledge about the risk factors of the disease, as well as our understanding of the effectiveness of successful therapeutic interventions. While a large number of risk factors for CVD have been identified in epidemiological studies, such as blood pressure, plasma lipid levels, diabetes, and smoking, familial studies and studies on twins have shown that about 40-50% of the susceptibility to CVD corresponds to the genetic factors 3. Several genetic risk factors associated with CVD have been identified in several studies, including mutations in *CYP4F2, ApoE,* and *CYP2A6* 4-7. Genetic analysis and mutational genotyping could potentially improve patients' outcomes if treatment is initiated early. However, more exploration is required.

The *CYP4F2* gene is a member of the CYP450 superfamily. It encodes an enzyme that plays a key role in the metabolism of endogenous and exogenous molecules such as vitamins K and E 8. As well, this enzyme is responsible for the metabolism of 20-hydroxy-eicosan-arachidonic acid (20-HETE). In rat hypertensive models, the increase in 20-HETE can cause oxidative stress and endothelial cell damage, which in turn increases the incidence of ischemic stroke (IS) 9. Recent studies in the Chinese population have shown that CYP4F2 is associated with an increased risk of IS 10. On the other hand, the *ApoE* gene located on chromosome 19 encodes an enzyme that plays an important role in lipoprotein metabolism. It facilitates the binding of lipoproteins to LDL receptors. However, defects in *ApoE* protein can reduce the ability of lipoproteins to bind to receptors, leading to high levels of cholesterol in the blood 11. Several studies have shown that *ApoE* polymorphisms are related to fluctuations in blood cholesterol. Therefore, they increase the risk of premature atherosclerosis and CVD, especially for coronary heart disease 12. The human cytochrome P450 gene (*CYP2A6*) encodes an enzyme that is predominantly expressed in the liver and is responsible for the metabolism of several clinically relevant substrates 13, 14. However, the enzymatic activity of *CYP2A6* and its metabolism varies greatly among individuals. The genetic variation of the *CYP2A6* gene is considered an important predictor of its activity 15.

One of the most commonly used anticoagulants for the treatment and prevention of thromboembolic disorders is warfarin 16. It consists of a mixture of R and S isomers, with the S isoform having an activity two to five times greater than the R isoform. The cytochrome P450 2C9 enzyme (*CYP2C9*) is the main system responsible for the metabolism of the S isoform in the liver 16, 17. Warfarin acts as an anticoagulant by binding to subunit 1 of vitamin K epoxide reductase (*VKORC1*), an enzyme required for the coagulation factors activation 18. In order to keep warfarin therapy safe and accurate, prothrombin time should be maintained at a therapeutic range, characterized by the International Normalized Ratio (INR) 19.

Moreover, achieving therapeutic INR may take weeks and can increase the risk of adverse drug reactions in the early stages of therapy 20. Thus, knowing how genetic and environmental factors influence the anticoagulant effect of warfarin will help in predicting the proper individualized/ personalized doses and leading to more accurate and safer treatment 21-23. Polymorphisms in the genes encoding drug receptors, metabolic enzymes, and transporters may regulate the response to warfarin 24. Similarly, a pharmacogenetics analysis revealed that genetic variations in the *CYP2C9* and *VKORC1* genes significantly affected the individual response to warfarin 25-27. Until now, there is no specific way to determine the exact dose of warfarin to reduce the risk of bleeding. Therefore, it is valuable to increase our understanding of the candidate genes associated with the oral anticoagulation pathway 28. The aim of the present study was to evaluate the genotypic associations between the selected SNPs in the *CYP4F2*, *ApoE,* and *CYP2A6* genes and the variability of the warfarin dose in Jordanian cardiovascular patients. As well, those genetic variations were assessed for their association with the risk factors and the susceptibility to CVDs.

## Materials and Methods

### Subjects

This study was ethically approved by the Royal Medical Services of Amman and the Human Research Committees of the Jordan University of Science and Technology in Irbid. Patients with written consent from the Queen's Alia Heart Institute Anticoagulation Clinics in Amman (a referral heart center for the peripheral military centers in Jordan) were enrolled in this study. This study excluded patients who received *CYP2C9* inducer or inhibitor medications, those who received concomitant medications that interact with, those who lost clinical data, patients known for alcohol consumption, and pregnant women. A total of 425 subjects participated in this study; non-cardiovascular health controls (n = 213) and cardiovascular patients (n = 212) over 18 years of age who had been receiving warfarin for at least 3 months.

### Data collection and follow-up time

Blood samples were collected to determine the value of INR and to genotype selected SNPs. Demographic characteristics include (body mass index, age, and gender), indications for warfarin treatment, a weekly average of INR value, a weekly average of warfarin dose (dose required to obtain therapeutic INR), medical problems, and concomitant medications were recorded during the patient clinic visits. Warfarin dose and INR values were recorded in two phases, the first phase is the beginning of the treatment where the INR values are unstable and is called (initiation phase of the therapy), and the second phase is when the INR values are stable in the therapeutic range for at least three sequential clinic visits, this stage is called (stabilization phase of therapy).

### Outcome measure

This study was divided into two main categories: a genetic association study (comparing the selected polymorphism between patients and control) and a pharmacogenetics study (examining the influence of the selected polymorphisms on warfarin treatment during the initiation and stabilization phases of therapy). The pharmacogenetics study addresses two main concepts. Warfarin sensitivity was the first concept: patients in this group were defined as either warfarin-resistant patients, who require high levels of warfarin dose to achieve therapeutic INR (> 49 mg/week), or normal patients, who require moderate doses ( 21-49 mg/week) or finally as sensitive patients who require a low dose (<21 mg/week). The later definition was based on the Gordon study (2009) 29. The second concept included warfarin responses and was evaluated according to the study by Higashi et al. (2002) in patients with good response (INR value within the therapeutic range), poor response (INR value below the target range), and ultra-response (INR above the target range) 30.

### SNP selection and genotyping

Candidate SNPs were selected from a public database such as the National Center for Biotechnology Information (NCBI) (http://www.ncbi.nlm.nih.gov/SNP/) and the Applied Biosystems SNP database (http://www.appliedbiosystems). Five SNPs were selected from the genes *CYP4F2*, *ApoE,* and *CYP2A6*. **Table S1** listed those genes, SNP IDs, and their locations. The DNA was extracted from the blood sample with the Wizard Genomic DNA Purification Kit (Promega), then samples that met the quantitative requirements were sent to the Australian Genome Research Facility (AGRF) to test the genotype using the MassARRAY_ system (iPLEX GOLD) (Sequenom, San Diego, CA, USA). The MassARRAY^TM^ system protocol and the primer information used for the *CYP4F2*, *ApoE*, and *CYP2A6* genes are available upon request.

### Statistical analysis

To examine which of the selected polymorphisms was associated with this susceptibility to CVDs and warfarin responses, a multivariate analysis was performed, which included a chi-squared test, one-way ANOVA, Tukey HSD post hoc multiple comparison test, and non-parametric correlation test. SPSS version 21.0 was used for all analyzes.

## Results

### Patient characteristics

A total of 212 cardiovascular patients with warfarin treatment and 213 healthy volunteers from Jordan were included in this study. Those participants were genetically tested and clinically evaluated to assess both the association between *CYP4F2*, *ApoE*, and *CYP2A6* polymorphisms and the susceptibility to cardiovascular disease and the impact of those polymorphisms on the sensitivity and responsiveness of warfarin during the initiation phase of therapy. Only 139 (66%) of the patients reached the stabilization phase and, they were used to assess the genetic effects of those polymorphisms on the sensitivity and responsiveness of warfarin during the stabilization phase of therapy. In total, 69.6% of patients were found to be good metabolizers, 15.2% were extensive metabolizers and 15.2% were poor metabolizers. Patients' demographics and clinical characteristics for each group were listed in a previous study by AL-Eitan et al. (2019) 31.

### Genotypic and allelic frequencies

One of the five selected SNPs was not polymorphic (Monomorphic) (rs11083750) from *ApoE* gene and only four SNPs were included in this study. The latter passed the quality control tests with high accuracy and a low rate of discordance. **Table S2** summarizes minor allelic frequencies and Hardy Weinberg equilibrium (HWE) p-values for the 212 cardiovascular patients and the 213 healthy controls. The genotypic frequencies of the *CYP4F2*, *ApoE,* and *CYP2A6* genes in the patients and controls are shown in **Table 1**. Only the rs7412 SNPs of the *ApoE* gene (CC/TC/TT) and (CC (TC + TT)) were significantly different between patients and controls with (*P-value* = 0.04) and (*P-value* = 0.02), respectively. In addition, significant associations were found between *ApoE* haplotypes (TCG) and cardiovascular disease in patients and healthy controls with (*P-value* <0.01) (**Table S3**).

### Association of *CYP4F2*, *ApoE,* and *CYP2A6* polymorphisms with warfarin sensitivity during the initiation and stabilization phases of therapy

Patients were defined as sensitive, moderate, and resistant patients, depending on the dose required to reach the therapeutic INR. All SNPs showed no significant difference between the three warfarin-sensitive groups during the initiation phase of therapy with (P > 0.05), except that the *CYP2A6* (rs1801272) sensitive group showed a high frequency of (TA) genotype (60%) while wild-type (AA) was (14%) with (overall *P-value* = 0.02) (**Table 2**). However, during the stabilization phase, no significant associations were found for all selected SNPs among the three sensitive groups with (*P-value* > 0.05) (**Table 3**). Furthermore, no significant association was observed between *ApoE* haplotypes and warfarin sensitivity (*P-value* > 0.05) (**Table S4**). There were no statistically significant differences between the doses required to reach the target INR during the initiation and stabilization phases of the treatment and the selected SNP genotypes (P > 0.05) with the exception of (rs7412) of the *ApoE* gene (*P-value* = 0.02) (**Table 4**). Likewise, post-hock tests do not show a significant association between SNPs and the required warfarin dose (*P-value* ≤ 0.05) (**Table S5**).

### Association of *CYP4F2*, *ApoE,* and *CYP2A6* polymorphisms and warfarin responsiveness during initiation and stabilization phases of therapy

According to the response to warfarin, the patients were divided into three groups: poor, good, and ultra-responders. There were no significant differences between the frequencies of the different genotypes in the three groups, in the initial phase of therapy with (*P-value* > 0.05) (**Table 5**). However, a significant association was found with the genetic polymorphism (rs1801272) of the *CYP2A6* gene during the stabilization phase of therapy, with 50% of the (TA) genotype were poor responders and only 5.2% of the wild-type (AA) were poorly responders (*P-value* = 0.002). In addition, (rs2108622) *CYP4F2* SNP showed a significant association between the distribution of the (CT) genotype among the three responder groups (*P-value* = 0.04) (**Table 6**), While no significant associations were found between the *ApoE* genetic haplotypes and the ability to respond to warfarin (*P-value* > 0.05) (**Table S6**). There were no significant differences between the selected SNPs and the INR values that ​​were measured in the initial phase of therapy in the 212 patients receiving warfarin (*P-value* > 0.05). However, differences were found between *CYP2A6* (rs1801272) and the INR values ​​that were measured in the 139 patients during the stabilization phase with (overall *P-value* = 0.02) (**Tables 7 and S7**).

## Discussion

In the current study, we examined and characterized the relationship between polymorphisms of the *CYP4F2*, *ApoE,* and *CYP2A6* genes and the increased risk of cardiovascular diseases, in addition to their relation with the sensitivity and responsiveness to warfarin during the initiation and stabilization phases of therapy in a Jordanian population. Regarding the relationship between those polymorphisms and the susceptibility to cardiovascular diseases, only one of the four investigated SNPs, (rs7412) *ApoE* gene, displayed significant association. According to our results, it was found that 9.7% of patients with CVDs were homogeneous or heterogeneous of the mutant allele (T) compared to only 3.8% in the control group. Many reports support the effect of this SNP on increasing the susceptibility for CVD. For instance, it was associated with increased BMI and Waist Girth 32. As well, it was associated with increased levels of LDL-C and TC 33.

In comparison to other studies, which focused mainly on the *ApoE* haplotype (rs7412, rs429358) instead of individual SNPs in the *ApoE* gene, the study of Lahoz et al (2011) showed that variations of the *ApoE* E2 (represented by rs7412 (T) allele and rs429358 (T) allele) and E4 (represented by rs7412 (C) allele and rs429358 (C) allele in the gene compared to the E3: E3 genotype may play a gender-specific role in CVD risk, with an increased risk of disease in men with E2 and E4 alleles 12.

Otherwise, Saudi and Taiwanese studies did not expose a statistically significant difference in the distribution of genotypes or alleles between patients with CVD and control groups according to the E2 *ApoE* haplotype. The Saudi E2 was closely associated with classic predictors of atherosclerosis 34, 35. In disagreement with our findings, the study by Viitanen et al. (2001) indicated that there is an increased risk of CVD in patient's carriers of the (T) variant allele of the rs405509 gene polymorphism, while our study did not show a significant association between this SNP and the risk of CVD 36. Moreover, our result didn't show significant differences in the distribution of genotypic frequency of rs2108622 *CYP4F2* SNP between patients and control and this finding was inconsistent with the result of Yan et al. (2015) which found that the variant allele of rs2108622 was significantly higher in the patient with IS than in the control group 10. Possibly, the differences in ethnicities, the underlying ischemic diseases, the disease stages, and other factors may contribute to such differences.

Regarding the pharmacogenetic part of this study, numerous warfarin pharmacogenetic studies indicated that the common polymorphisms of *VKORC1* and *CYP2C9* together with environmental and clinical factors account for more than half of the variation in the required warfarin dose 37, 38. We have recently published a study examining the effects of genetic polymorphism of *CYP2C9*, *VKORC1, FVII, PSRC1, CELSR2, and SORT1* on the sensitivity and responsiveness to warfarin treatment during the initiation and stabilization phase of therapy in Jordanian Arab cardiovascular patients 26, 27, 39, 40. In this study, our goal was to expand our knowledge and identify other genetic factors associated with warfarin sensitivity and response, including the *CYP4F2, ApoE,* and *CYP2A6* genes. The results of the current pharmacogenetic study strongly suggest that there is a significant association of *CYP2A6* rs1801272 SNP with warfarin sensitivity and between *ApoE* rs7412 SNP and the variability of the warfarin required dose during the initiation phase of therapy. This study also reported that there is a genetic association between *CYP2A6* rs1801272 SNP and the ability to respond to warfarin and INR measurements during the stabilization phase of therapy. Few studies reported a direct effect of those SNPs on warfarin metabolism. For instance, according to some reports, the *CYP2A6* (which is sometimes called a human coumarin 7-hydroxylase) rs1801272 SNP was associated with no activity effect on the protein and therefore can modulate its effect on the warfarin metabolism 41. On the other hand, other studies from the region reported no effect of the APOE SNPs including the rs7412 SNP in warfarin dose adjustment 42 while other Asian studies on Chinese people showed positive associations 43.

The minor allele frequency of *CYP4F2* rs2108622 SNP in our population was 38%, which is similar to that found in white and Asian ethnic groups, while it differs from the black ethnic group with only 7% 44. According to Jarrar et al., a high frequency of CYP4F2 rs2108622 SNP was observed among healthy Arab Jordanians 45. However, Caldwell et al (2019) study revealed that patients carrier for (T) variant allele decrease the *CYP4F2* enzyme activity and therefore lead to an increase in the warfarin required dose 44. Moreover, a study in the Japanese population showed that the average daily warfarin dose in the *CYP4F2* (CT/ TT) carriers were 28.2% higher than in the *CYP4F2* (CC) wild-type carriers 46. Accordingly, our finding showed that the patient homozygous for the variant allele (TT) had required a weakly warfarin dose of 44.59 [49.83] mg compared to 35.87 [15.45] mg/weak for the wild-type (CC) allele during the initial stages of the therapy, but this was not statistically significant (**Table 4**). Moreover, when comparing the three sensitive groups (sensitive, moderate, and resistant) during the stabilization phase of the therapy, our results also implicated that 31.6% of the resistance group were (TT) carriers and 25.4% were (CT) carriers in comparison to 19.7% of them who were (CC) carriers for wild-type allele, therefore patients with (TT) and (CT) polymorphisms needed higher doses of warfarin to achieve their therapeutic effect, but again this was not statistically significant (**Table 3**). However, when comparing the three responder groups (poor, good, and ultra), this SNP showed no differences between the three responder groups and with the INR measurements during the initiation and stabilization phase of the therapy, which was consistent with the Japanese study, indicating that the mean PT-INR values ​​were not differentiated between the *CYP4F2* genotypes 46.

As mentioned earlier, most studies focused mainly on the *ApoE* haplotype (rs7412, rs429358) instead of individual SNPs in the *ApoE* gene. However, although some studies showed a significant association between the allelic variant *ApoE* E4 and the higher dose of warfarin 24, 47, other studies have demonstrated that there is no association between warfarin dose and *ApoE* variants 48-50. In addition, a study in the Egyptian population showed that *ApoE* E2 variants were significantly associated with the lowest warfarin dose 51. In this study, we observed that the *ApoE* (T) allele (rs7412) at the initiation of treatment was significantly associated with higher warfarin dose requirements, the patient with the (CT) allele received 53.39 [62.10] mg/weak compared to 36.79 14,33 mg/weak in (CC) carriers (P-*value* = 0.02) (**Table 3**).

However, there were no significant differences between this SNP and the sensitivity or responses to warfarin during the initiation or stabilization phase of therapy. According to the literature on *ApoE* gene, there was a significant relationship between re405509 and susceptibility to CVD, Alzheimer's disease, and obstructive sleep apnea in children 36, 52, 53, but to our knowledge, no study was conducted to evaluate the effect of this SNP on the warfarin sensitivity and responsiveness. Again, our result showed no significant difference between this SNP and the sensitivity or the response to warfarin during the initial or stabilization phase of therapy. The frequency of minor alleles of *CYP2A6* rs1801272 SNP in our population was 1.5%, which was consistent with that found in African-American and Brazilian ethnic groups with 1.1% and 1.7%, respectively, and it was less than what was found in the Chinese population with 2.2% and more than Egyptian and Japanese population with 0.7% and 0.0%, respectively 54. According to the study conducted in the Canadian population, patients with allelic polymorphisms (T) variants are associated with a greater sensitivity to warfarin and, therefore, lead to a significant reduction in the weekly dose of warfarin 55. Correspondingly, in our study, a significant association was observed between this SNP and warfarin sensitivity, with 60% of warfarin-sensitive patients were heterozygous for the variant allele (TA) at the initiation of therapy (*P-value* = 0.02) (**Table 2**) and therefore, these patients required 33.64 [34.87] mg/weak of warfarin dose compared to 38.16 [22.83] mg/weak for wild-type carriers (**Table 4**). Furthermore, our result showed a significant difference between this SNP and the responsiveness to warfarin during the stabilization phase of therapy with 50% of poor responder patients were heterozygous for the variant allele (TA) and therefore the INR values have differed significantly between the (TA) carrier patients and the (AA) wild-type with (*P-value* = 0.02) (**Tables 6 and 7**). This result may be explained by the effect of this variant in substantially reducing the activity of the CYP2A6 enzyme 56. However, we cannot anticipate the exact effect of this reduction on the enzymatic metabolism of warfarin, especially that warfarin can be metabolized through other pathways and the effect can be compensated by the action of other enzymes.

## Conclusion

Our data show a significant association between the *ApoE* rs7412 SNP and the risk of CVD in the Jordanian population. Moreover, *ApoE* rs7412 SNP exhibited significant differences in warfarin initial doses. As well, a significant association was found between the *CYP2A6* rs1801272 polymorphism and warfarin sensitivity during the initiation phase of therapy and with warfarin responsiveness and INR measurement during the stabilization phase of therapy. To confirm our results, further studies with a large number of samples and different populations are required. Personalized warfarin prescription can be a safe and effective anticoagulation therapy for patients with cardiovascular disease. Therefore, more pharmacogenetic studies are needed to evaluate the effects of other clinical and genetic factors, thereby facilitating the prevention and treatment of cardiovascular disease.

## Supplementary Material

Supplementary tables.

## Figures and Tables

**Table 1:** The distributions of *CYP4F2*, *APOE* and *CYP2A6* SNPs in 212 cardiovascular patients and 213 healthy controls

Gene	SNP ID	Model	Patients %	Controls %	*P*-value*
*CYP4F2*	rs2108622	CC/CT/TT	43.9/42.5/13.7	35.2/46.5/18.3	0.15
		CC/(CT+TT)	43.9/56.1	35.2/64.8	0.07
		(CC+CT)/TT	86.3/13.7	81.7/18.3	0.19
*APOE*	rs7412	CC/TC/TT	90.3/9.2/0.5	96.2/3.8/0	0.04
		CC(TC+TT)	90.3/9.7	96.2/3.8	0.02
		(CC+TC)/TT	99.5/0.5	100/0	0.24
*APOE*	rs405509	GG/GT/TT	31.6/43.4/25	27.2/51.2/21.6	0.28
		GG/(GT+TT)	31.6/68.4	27.2/72.8	0.32
		(GG+GT)/TT	75/25	78.4/21.6	0.41
*CYP2A6*	rs1801272	AA/TA	97.6/2.4	96.2/3.8	0.39

*Chi-Square Test with *p* < 0.05 is considered significant.

**Table 2:** Associations of *CYP4F2*, *APOE* and *CYP2A6* SNPs with warfarin sensitivity during the initiation phase of therapy of 212 cardiovascular patients treated with warfarin

Gene	SNP ID	Genotype	Sensitive^a^	Moderate^b^	Resistance^c^	Overall *P*-value*
*CYP4F2*	rs2108622	CC	(19/93) 20.4%	(62/93) 66.7%	(12/93) 12.9%	0.18
		*P*-value*	0.16	0.83	0.55	
		CT	(10/90) 11.1%	(61/90) 67.8%	(19/90) 21.1%	
		*P*-value*	0.38	0.96	0.22	
		TT	(3/29) 10.3%	(23/29) 79.3%	(3/29) 10.3%	
		*P*-value*	0.74	0.43	0.67	
*APOE*	rs7412	CC	(28/187) 15%	(129/187) 69%	(30/187) 16%	0.94
		*P*-value*	1	0.95	0.9	
		TC	(3/19) 15.8%	(12/19) 63.2%	(4/19) 21.1%	
		*P*-value*	1	0.86	0.86	
		TT	(0/1) 0.0%	(1/1) 100%	(0/1) 0.0%	
		*P*-value*	0.90	0.78	0.9	
*APOE*	rs405509	GG	(13/67) 19.4%	(43/67) 64.2%	(11/67) 16.4%	0.59
		*P*-value*	0.49	0.61	1	
		GT	(12/92) 13.0%	(63/92) 68.5%	(17/92) 18.5%	
		*P*-value*	0.77	1	0.70	
		TT	(7/53) 13.2%	(40/53) 75.5%	(6/53) 11.3%	
		*P*-value*	0.90	0.49	0.56	
*CYP2A6*	rs1801272	AA	(29/207) 14%	(145/207) 70%	(33/207) 16%	0.02
		*P*-value*	0.02	0.06	0.97	
		TA	(3/5) 60%	(1/5) 20%	(1/5) 20%	
		*P*-value*	0.02	0.06	0.97	

*Chi-square test with *p* value < 0.05 is considered significant. a Warfarin Sensitive group (required minimum warfarin dose < 21 mg/week). b Warfarin Moderate response group (required average warfarin dose between 21 and 49 mg/week). c Warfarin Resistance group (required high warfarin dose > 49 mg/week).

**Table 3:** Associations of *CYP4F2*, *APOE* and *CYP2A6* SNPs with warfarin sensitivity during the stabilization phase of therapy of 139 cardiovascular patients treated with warfarin

Gene	SNP ID	Genotype	Sensitive^a^	Moderate^b^	Resistance^c^	Overall *P*-value*
*CYP4F2*	rs2108622	CC	(10/61) 16.4%	(39/61) 63.9%	(12/61) 19.7%	0.66
		*P*-value*	0.84	0.90	0.61	
		CT	(9/59) 15.3%	(35/59) 59.3%	(15/59) 25.4%	
		*P*-value*	0.97	0.87	0.92	
		TT	(1/19) 5.3%	(12/19) 63.2%	(6/19) 31.6%	
		*P*-value*	0.47	0.99	0.69	
*APOE*	rs7412	CC	(17/122) 13.9%	(76/122) 62.3%	(29/122) 23.8%	0.67
		*P*-value*	0.68	0.84	1	
		TC	(3/13) 23.1%	(7/13) 53.8%	(3/19) 23.1%	
		*P*-value*	0.68	0.84	1	
*APOE*	rs405509	GG	(8/42) 19%	(29/42) 69%	(5/42) 12%	0.24
		*P*-value*	0.1	0.52	0.59	
		GT	(7/61) 11.5%	(35/61) 57.4%	(19/61) 31.1%	
		*P*-value*	0.69	0.63	0.19	
		TT	(5/36) 13.9%	(22/36) 61.1%	(9/36) 25%	
		*P*-value*	1	1	0.98	
*CYP2A6*	rs1801272	AA	(18/135) 13.3%	(85/135) 63%	(32/135) 23.7%	0.10
		*P*-value*	0.12	0.31	1	
		TA	(3/4) 50%	(1/4) 25%	(1/4) 25%	
		*P*-value*	0.12	0.31	1	

*Chi-square test with *p* value < 0.05 is considered significant. a Warfarin Sensitive group (required minimum warfarin dose < 21 mg/week). b Warfarin Moderate response group (required average warfarin dose between 21 and 49 mg/week). c Warfarin Resistance group (required high warfarin dose > 49 mg/week).

**Table 4:** Association of *CYP4F2*, APOE and *CYP2A6* SNPs with variability on warfarin required doses

Gene	SNP ID	Genotype	Initiation dose	Overall *P*-value*	Maintenance dose	Overall *P*-value*
		CC	35.87 [15.45]		36.66 [17.11]	
*CYP4F2*	rs2108622	CT	38.22 [14.67]	0.21	40.24 [19.99]	0.54
		TT	44.59 [49.83]		39.43 [12.76]	
		CC	36.79 [14.33]		38.24 [17.24]	
*APOE*	rs74125	TC	53.39 [62.10]	0.02	38.60 [25.09]	0.48
		TT	38.40 […….]			
		GG	38.13 [37.72]		35.49 [17.74]	
*APOE*	rs405509	GT	38.28 [15.55]	0.98	41.01 [18.36]	0.30
		TT	37.60 [14.39]		37.98 [17.04]	
*CYP2A6*	rs1801272	AA	38.16 [22.83]	0.67	38.56 [16.79]	0.99
		TA	33.64 [34.87]		38.60 [45.86]	

*One-way ANOVA test with *P*-value < 0.05 is considered significant, Mean Standard deviation in square brackets.

**Table 5:** Association of *CYP4F2*, *APOE* and *CYP2A6* SNPs with warfarin responsiveness during the initiation phase of therapy of 212 cardiovascular patients treated with warfarin

Gene	SNP ID	Genotype	Poor responders^a^	Good responders^b^	Ultra responders^c^	Overall *P*-value*
*CYP4F2*	rs2108622	CC	(17/93) 18.3%	(72/93) 77.4%	(4/93) 4.3%	0.54
		*P*-value*	1	0.95	0.88	
		CT	(17/90) 18.9%	(66/90) 73.3%	(7/90) 7.8%	
		*P*-value*	0.99	0.66	0.34	
		TT	(5/29) 17.2%	(24/29) 82.8%	(0/29) 0.0%	
		*P*-value*	0.99	0.69	0.40	
*APOE*	rs7412	CC	(34/187) 18.2%	(143/187) 76.5%	(10/187) 5.3%	0.25
		*P*-value*	0.76	0.99	0.57	
		TC	(4/19) 21.1%	(15/19) 78.9%	(0/19) 0.0%	
		*P*-value*	0.97	0.96	0.59	
		TT	(1/1) 100%	(0/1) 0.0%	(0/1) 0.0%	
		*P*-value*	0.11	0.20	0.98	
*APOE*	rs405509	GG	(14/67) 20.9%	(51/67) 76.1%	(2/67) 3%	0.55
		*P*-value*	0.81	1	0.62	
		GT	(16/92) 17.4%	(72/92) 78.3%	(4/92) 4.3%	
		P-value*	0.95	0.86	0.89	
		TT	(9/53) 17%	(39/53) 73.6%	(5/53) 9.4%	
		*P*-value*	0.96	0.86	0.27	
*CYP2A6*	rs1801272	AA	(37/207) 17.9%	(159/207) 76.8%	(11/207) 5.3%	0.42
		*P*-value*	0.45	0.68	0.87	
		TA	(2/5) 40%	(3/5) 60%	(0/5) 0%	
		*P*-value*	0.45	0.68	0.87	

*Chi-Square Test with *p* value < 0.05 is considered significant. a Poor responders (INR value below target). b Good responders who have an INR value in the target range (therapeutic range). c Ultra responders (INR over target).

**Table 6:** Association of *CYP4F2*, *APOE* and *CYP2A6* SNPs with warfarin responsiveness during the stabiliaiztaion phase of therapy of 139 cardiovascular patients treated with warfarin

Gene	SNP ID	Genotype	Poor responder^a^	Good responder^b^	Ultra responder^c^	Overall *P*-value*
		CC	(2/61) 3.3%	(57/61) 93.4%	(2/61) 3.3%	0.12
*CYP4F2*	rs2108622	*P*-value*	0.40	0.36	0.87	
		CT	(7/59) 11.9%	(48/59) 81.4%	(4/59) 6.8%	
		*P*-value*	0.09	0.04	0.47	
		TT	(0/19) 0%	(19/19) 100%	(0/19) 0%	
		*P*-value*	0.47	0.26	0.61	
		CC	(8/122) 6.6%	(108/122) 88.5%	(6/122) 4.9%	0.71
*APOE*	rs7412	*P*-value*	0.99	0.92	0.72	
		TC	(1/13) 7.7%	(12/13) 92.3%	(0/19) 0%	
		*P*-value*	0.99	0.92	0.72	
		GG	(3/42) 7.1%	(38/42) 90.5%	(1/42) 2.4%	
		*P*-value*	0.98	0.95	0.76	
*APOE*	rs405509	GT	(3/61) 4.9%	(56/61) 91.8%	(2/61) 3.3%	
		*P*-value*	0.80	0.68	0.87	0.64
		TT	(3/36) 8.3%	(30/36) 83.4%	(3/36) 8.3%	
		*P*-value*	0.87	0.42	0.39	
*CYP2A6*	rs1801272	AA	(7/135) 5.2%	(122/135) 90.4%	(6/135) 4.4%	0.002
		*P*-value*	<0.0001	0.04	0.91	
		TA	(2/4) 50%	(2/4) 50%	(0/4) 0%	
		*P*-value*	<0.0001	0.04	0.91	

*Chi-Square Test with *p* value < 0.05 is considered significant. a Poor responders (INR value below target). b Good responders who have an INR value in the target range (therapeutic range). c Ultra responders (INR over target).

**Table 7:** Association of *CYP4F2*, APOE and *CYP2A6* SNPs with INR treatment outcome

Gene	SNP ID	Genotype	Initiation INR	Overall *P*-value*	Maintenance INR	Overall *P*-value*
		CC	2.47 [0.77]		2.69 [0.38]	
*CYP4F2*	rs2108622	CT	2.50 [0.81]	0.34	2.70 [0.41]	0.97
		TT	2.27 [0.61]		2.69 [0.35]	
		CC	2.45 [0.75]		2.71 [0.39]	
*APOE*	rs74125	TC	2.37 [0.79]	0.29	2.49 [0.34]	0.06
		TT	1.30 […...]			
		GG	2.45 [0.83]		2.69 [0.43]	
*APOE*	rs405509	GT	2.43 [0.78]	0.82	2.69 [0.36]	0.99
		TT	2.51 [0.69]		2.70 [0.40]	
*CYP2A6*	rs1801272	AA	2.44 [0.77]	0.09	2.71 [0.38]	0.02
		TA	3.02 [0.81]		2.25 [0.44]	

*One-way ANOVA test with *P*-value < 0.05 is considered significant, Mean Standard deviation in square brackets.
