# Supplementary material for: Influence of CYP4F2, ApoE, and CYP2A6 gene polymorphisms on the variability of Warfarin dosage requirements and susceptibility to cardiovascular disease in Jordan
Source: Int J Med Sci. 2021 Jan 1;18(3):826–34. doi: 10.7150/ijms.51546 (PMC7797549; doi:10.7150/ijms.51546)
Supplement: Supplementary file 1 — Supplementary tables. [file ijmsv18p0826s1.pdf]

**Table S1** Genes and SNPs characteristics

| Gene | SNP ID | Chr Position[a] | SNP | SNP Type |
|---|---|---|---|---|
| *CYP4F2* | rs2108622 | 19:15879621 | C/T | Missense Variant |
| *APOE* | rs7412 | 19:44908822 | C/T | Missense Variant |
| *APOE* | rs11083750 | 19:44908601 | C/A<br>C/G<br>C/T | Missense Variant |
| *APOE* | rs405509 | 19:44905579 | T/G | 2KB Upstream Variant |
| *CYP2A6* | rs1801272 | 19:40848628 | A/T | Missense Variant |

a. Chromosome positions are based on NCBI Human Genome Assembly Build.

**Table S2** List of SNPs, their minor allele frequencies, and HWE p-values

| Gene | SNP ID | MA[a] | Patients MAF[b] | Controls MAF[b] | HWE[c] P-value |
|---|---|---|---|---|---|
| *CYP4F2* | rs2108622 | T | 0.35 | 0.42 | 0.26 |
| *APOE* | rs7412 | T | 0.05 | 0.02 | 0.4 |
| *APOE* | rs405509 | T | 0.47 | 0.47 | 0.33 |
| *CYP2A6* | rs1801272 | T | 0.01 | 0.02 | 1 |

a. MA: Minor allele.
b. MAF: Minor allele frequency.
c. HWE: Hardy-Weinberg equilibrium.

**Table S3** The distributions of *APOE* haplotype and 211 cardiovascular patients in compare to 213 healthy controls

| Gene | Haplotypes | Patients (%) | Controls (%) | Odds ratio (95% CI) | P-value* |
|---|---|---|---|---|---|
| *APOE* | CCG | 0.48 | 0.51 | 1.00 | --- |
| | CCT | 0.47 | 0.47 | 1.08 (0.82 - 1.42) | 0.58 |
| | TCG | 0.05 | 0.02 | 3.53 (1.36 - 9.21) | **0.01** |

*Chi–Square Test with $p<0.05$ is considered significant.

**Table S4** The distributions of *APOE* haplotype among 212 warfarin sensitive patients

| Gene | Haplotypes | Frequency (%) | Odds ratio (95% CI) | P-value* |
|---|---|---|---|---|
| *APOE* | CG | 0.50 | 0.00 | --- |
| | CT | 0.45 | -0.01 (-0.12 - 0.1) | 0.84 |
| | TG | 0.03 | -0.13 (-0.62 - 0.36) | 0.59 |
| | TT | 0.02 | 0.2 (-0.25 - 0.66) | 0.38 |

*Chi–Square Test with $p<0.05$ is considered significant.

**Table S5** Post Hoc tests for the Association of *CYP4F2*, *APOE* and *CYP2A6* SNPs with variability on warfarin required doses

| Gene | SNP ID | Genotype | | Initiation Dose P-value* | Maintenance Dose P-value* |
|---|---|---|---|---|---|
| *CYP4F2* | rs2108622 | CC | CT | 0.77 | 0.52 |
| | | | TT | 0.18 | 0.83 |
| | | CT | CC | 0.77 | 0.52 |
| | | | TT | 0.40 | 0.98 |
| | | TT | CC | 0.18 | 0.52 |
| | | | CT | 0.40 | 0.98 |
| *APOE* | rs405509 | GG | GT | 1 | 0.27 |
| | | | TT | 0.99 | 0.81 |
| | | GT | GG | 1 | 0.27 |
| | | | TT | 0.98 | 0.70 |
| | | TT | GG | 0.99 | 0.81 |
| | | | GT | 0.98 | 0.70 |

* Post-Hock Multiple comparisons Test with $p<0.05$ is considered significant. Com initiation and maintenance dose among all genotypes.
Post hock are not performed for rs1801272 because its fewer than three groups.
Post hoc tests are not performed for rs74125because at least one group has fewer than two cases.

**Table S6** The distributions of *APOE* haplotypeamong 212 warfarin responsiveness patients

| Gene | Haplotypes | Frequency (%) | Odds ratio (95% CI) | P-value* |
|---|---|---|---|---|
| *APOE* | CG | 0.49 | 0.00 | --- |
| | CT | 0.46 | 0.04 (-0.05 - 0.13) | 0.39 |
| | TG | 0.05 | -0.15 (-0.37 - 0.07) | 0.18 |

*Chi–Square Test with p<0.05 is considered significant.

**Table S7** Post Hoc tests for the association of *CYP4F2*, *APOE* and *CYP2A6* SNPs with INR treatment outcome

| Gene | SNP ID | Genotype | | Initiation INR P-value* | Maintenance INR P-value* |
|---|---|---|---|---|---|
| *CYP4F2* | rs2108622 | CC | CT | 0.95 | 0.98 |
| | | | TT | 0.43 | 1 |
| | | CT | CC | 0.95 | 0.98 |
| | | | TT | 0.32 | 0.99 |
| | | TT | CC | 0.43 | 0.98 |
| | | | CT | 0.32 | 0.99 |
| *APOE* | rs405509 | GG | GT | 0.99 | 1 |
| | | | TT | 0.89 | 0.99 |
| | | GT | GG | 0.99 | 1 |
| | | | TT | 0.80 | 0.99 |
| | | TT | GG | 0.89 | 0.99 |
| | | | GT | 0.88 | 0.99 |

* Post-Hock Multiple comparisons Test with p<0.05 is considered significant. Compare means of the initiation and maintenance dose among all genotypes.

Post hock are not performed for rs1801272because its fewer than three groups

Post hoc tests are not performed for rs74125because at least one group has fewer than two cases.
